# The Role of Lipids and Lipoproteins in Health: Insights from a Special Issue

**DOI:** 10.3390/nu18121964

**Published:** 2026-06-18

**Authors:** Baohai Shao

**Affiliations:** Department of Medicine, University of Washington, Seattle, WA 98109, USA; bhshao@uw.edu

Lipids and lipoproteins are essential for human physiology, functioning not only as structural and energetic components but also as dynamic regulators of metabolic homeostasis and inter-organ communication [1,2]. Dysregulation of lipid metabolism contributes to a broad spectrum of cardiometabolic disorders, including cardiovascular disease (CVD), metabolic dysfunction-associated steatotic liver disease (MASLD), obesity, diabetes, and chronic kidney disease [3]. Traditionally, lipid research has focused largely on circulating cholesterol concentrations, particularly low-density lipoprotein cholesterol (LDL-C) and high-density lipoprotein cholesterol (HDL-C). However, increasing evidence indicates that lipoprotein functionality, apolipoprotein composition, tissue-specific lipid trafficking, and gene–environment interactions may provide deeper insight into disease mechanisms than conventional lipid measurements alone. Consistent with this evolving perspective, recent dyslipidemia guidelines increasingly recognize the importance of atherogenic lipoproteins beyond LDL-C alone, including triglyceride-rich lipoproteins, lipoprotein(a), and apoB-containing lipoproteins, reflecting the growing complexity of lipid-related cardiovascular risk assessment [4].

This Special Issue, “The Role of Lipids and Lipoproteins in Health”, brings together studies exploring the diverse biological and clinical roles of lipoproteins in metabolic and cardiovascular diseases. Collectively, the contributions highlight the growing transition from viewing lipoproteins simply as cholesterol carriers toward understanding them as metabolically active and functionally heterogeneous particles involved in inflammation, oxidative stress, ectopic fat deposition, and cardiometabolic risk regulation.

A major theme emerging from this Special Issue is the close relationship between lipoprotein metabolism and MASLD. MASLD, formerly known as non-alcoholic fatty liver disease (NAFLD), represents the most common chronic liver disease worldwide and is strongly associated with obesity, insulin resistance, dyslipidemia, and increased cardiovascular risk [5,6]. Altered triglyceride-rich lipoprotein metabolism, elevated LDL-C, and reduced HDL-C are common metabolic features of MASLD and contribute substantially to its systemic complications [7,8].

Two studies in this Special Issue addressed the relationship between HDL-related pathways and MASLD progression. Lioci et al. (contribution 1) investigated the effects of intestinal-specific activation of liver X receptor alpha (LXRα) in a murine MASLD/metabolic-associated steatohepatitis (MASH) model. The authors demonstrated that intestinal activation of LXRα increased circulating HDL levels and hepatic scavenger receptor class B type 1 (SR-B1) expression, accompanied by improvements in reverse cholesterol transport, inflammation, and fibrosis. Their findings further suggested a shift from pro-inflammatory toward anti-inflammatory macrophage phenotypes in liver tissue. This work supports the concept that tissue-specific modulation of lipid signaling pathways may represent a promising therapeutic strategy for MASLD and related metabolic disorders [9].

Martínez-Montoro et al. (contribution 2) evaluated the triglyceride-to-HDL-C ratio as a marker for biopsy-proven MASLD in patients with obesity. Because the TG/HDL-C ratio reflects both elevated triglycerides and reduced HDL-C, it has frequently been proposed as a surrogate marker of insulin resistance and cardiometabolic risk [10,11,12]. The authors observed significantly higher TG/HDL-C ratios in patients with MASLD; however, the ratio did not outperform triglyceride or HDL-C levels alone in identifying disease. These findings underscore both the potential and the limitations of simple lipid ratios as biomarkers in complex metabolic liver disorders.

Another important theme in this Special Issue concerns the relationship between obesity, anthropometric measurements, and lipid-associated cardiovascular risk. Epidemiological studies have consistently demonstrated strong associations between obesity-related indices and adverse lipid profiles [13]. Arroyo et al. (contribution 3) explored the Equation Córdoba for Estimation of Body Fat (ECORE-BF), an anthropometric index derived from age, sex, and body mass index [14], in a very large Spanish working population. The authors found that higher ECORE-BF values were associated with several atherogenic lipid indices, particularly atherogenic dyslipidemia and lipid triad phenotypes. Their findings suggest that simple anthropometric tools may help identify individuals with adverse lipid-related cardiovascular risk profiles in large-scale or resource-limited settings, although such indices cannot replace direct biochemical lipid measurements.

Genetic factors play important roles in obesity development and lipid metabolism, with obesity-associated polymorphisms influencing adipogenesis, insulin signaling, and cholesterol homeostasis [15]. Hernández-Guerrero et al. (contribution 4) investigated obesity-associated polymorphisms and their interactions with anthropometric variables in a Mexican population. The study demonstrated that genetic risk score (GRS) interactions with waist-to-hip ratio (WHR) significantly influenced LDL-C levels, supporting the concept that lipid phenotypes are shaped not only by genetic susceptibility but also by body fat distribution and environmental influences. Although the overall predictive power of the multivariate model remained modest, the findings emphasize the complexity of gene–environment interactions in dyslipidemia and cardiovascular risk.

Several contributions also highlighted the importance of lipoprotein functionality beyond cholesterol concentration alone. Excessive intake of high-fat and high-fructose diets contributes to oxidative stress, impaired reverse cholesterol transport [16], and dyslipidemia, partly through altered ATP-binding cassette transporter A1 (ABCA1) activity, a key regulator of HDL biogenesis and cholesterol efflux. Serafín-Fabián et al. (contribution 5) examined the effects of nicotinamide supplementation in a diet-induced dyslipidemia model. The authors demonstrated that nicotinamide improved HDL-C levels, enhanced hepatic antioxidant responses, and modulated hepatic ABCA1 expression. These findings suggest potential interactions between oxidative stress pathways and HDL metabolism, supporting further investigation of metabolic interventions targeting HDL functionality rather than HDL-C concentration alone.

The growing complexity of triglyceride-rich lipoprotein biology is illustrated by the work of Liu et al. (contribution 6), who investigated the relationship between very low-density lipoprotein cholesterol (VLDL-C) and intra-pancreatic fat deposition (IPFD). Excessive IPFD has emerged as an important metabolic abnormality associated with insulin resistance, diabetes, and cardiovascular risk [17]. The study demonstrated that the association between VLDL-C and IPFD was influenced by dysapolipoproteinemia involving apoB, apoC-II, and apoC-III. These findings reinforce the concept that apolipoprotein composition and lipoprotein metabolism may influence ectopic lipid accumulation in a tissue-specific manner and that cholesterol concentration alone may not fully reflect lipoprotein-related disease risk.

Collectively, the studies published in this Special Issue support several broader concepts that are reshaping modern lipid research. First, traditional lipid measurements such as LDL-C and HDL-C remain clinically important but are increasingly recognized as incomplete descriptors of lipoprotein biology. Lipoprotein composition, particle function, and metabolic context may be equally important determinants of disease risk. Second, tissue-specific lipid accumulation and organ-specific lipoprotein interactions—including in the liver and pancreas—play important roles in cardiometabolic disease progression. Third, obesity, body fat distribution, and genetic susceptibility interact in complex ways to influence lipid phenotypes and cardiovascular risk.

At the same time, several important challenges remain unresolved. Many currently proposed lipid biomarkers remain primarily associative, while the causal mechanisms linking altered lipoprotein metabolism to tissue injury are incompletely understood. In addition, advanced approaches such as lipidomics, lipoprotein proteomics, and functional HDL assays are not yet routinely integrated into clinical practice. Future research will likely require the integration of multi-omics technologies, tissue-specific metabolic studies, and longitudinal clinical investigations to better define how lipoprotein function contributes to disease development and therapeutic response.

In summary, the contributions in this Special Issue illustrate the continuing evolution of lipoprotein biology from a cholesterol-centered framework toward a broader and more integrative understanding of metabolic disease. Beyond serving as passive lipid carriers, lipoproteins are increasingly recognized as biologically active and functionally diverse regulators of inflammation, oxidative stress, lipid trafficking, and cardiometabolic homeostasis. Continued advances in this field may ultimately improve risk stratification and facilitate the development of more precise therapeutic strategies for metabolic and cardiovascular diseases.

## Abbreviations

The following abbreviations are used in this manuscript:

ABCA1	ATP-binding cassette transporter A1
ApoB	apolipoprotein B
ApoC-II	apolipoprotein C-II
ApoC-III	apolipoprotein C-III
CVD	cardiovascular disease
ECORE-BF	Equation Córdoba for Estimation of Body Fat
GRS	genetic risk score
HDL-C	high-density lipoprotein cholesterol
IPFD	intra-pancreatic fat deposition
LDL-C	low-density lipoprotein cholesterol
LXRα	liver X receptor alpha
MASH	metabolic-associated steatohepatitis
MASLD	metabolic dysfunction-associated steatotic liver disease
NAFLD	non-alcoholic fatty liver disease
SR-B1	scavenger receptor class B type 1
TG/HDL-C ratio	triglyceride-to-HDL-C ratio
VLDL-C	very low-density lipoprotein cholesterol
WHR	waist-to-hip ratio
